# Correspondence

**Published:** 1871-06

**Authors:** 


### Correspondence.

EDITOR CHICAGO MEDICAL JOURNAL.

My address to the last graduating class in Rush Medical College, has called out an article from the pen of Dr. McElroy, which he opens as follows:

"Questions of science and fact are not often settled by after-dinner addresses. And for their utterances on such occasions, gentlemen are very properly not held to very strict account.

"But it is, nevertheless, to be regretted that those whose positions in life give to their utterances on all public occasions an *ex cathedra* importance, should ever discuss questions of science and fact as partisans of particular conclusions, in the absence of positive demonstration.

"Prof. Gunn, at the recent commencement of Rush Medical College, discussed at some length, and in no catholic and scientific spirit, it seems to me, the correlation of the physical and vital forces; but rather as a partisan that no such correlation exists in fact."

The Doctor then proceeds to give a history of his own thoughts, reasoning, and conclusions upon the subject. I read his article with care and interest, but I do not propose to reply to any portion of it, save that which I have quoted, further than to remark that it is unfortunate that it was not an after-dinner effort, so as to be entitled to that leniency, which the Doctor, himself, says should be awarded to such productions; for surely, an article which persistently denies the existence of a vital force and yet strenuously labors to correlate it with physical forces is in sore need of charity.

I am charged with an uncatholic, unscientific and partisan spirit in discussion. I regret that I have incurred the application of the first two adjectives; as to the third, if an earnest effort to expose the fallacies of the new philosophy earns me the title of partisan, I accept it with all cheerfulness; but my partisanship will not prevent my recognizing and accepting truth whenever and wherever presented. As to the charge that there was an absence of demonstration, a reference to my address will show that the several positions that I took were demonstrated by a reference to facts which are not denied by any one. Neither my positions nor facts have been directly assaulted by Dr. McElroy, but he has given them a wide berth. Whenever they are attacked I will endeavor to defend them, unless I am convinced that they are unworthy of defense, in which event I will confess my error.

MOSES GUNN.

---

MATTOON, ILL., April 25, 1871.

EDITORS CHICAGO MEDICAL JOURNAL:

The following treatment for paraphymosis is, as far as I know, entirely new, but has been entirely successful in my hands, and as these cases are sometimes very annoying to the young practitioner, and as (most persons are very nervous about the use of the knife on so important an organ) they are frequently compelled to send for consultation, I send the following for their benefit.

The patient standing erect, first pour a stream of cold water (ice water, if at hand,) upon the glans penis for a minute or two steadily, then grasp the glans firmly between the thumb and index finger of the left hand, and make pretty forcible traction in a direction at right angles to the body of the patient, pressing firmly upon the glans at the same time, while with the thumb and index finger of the right hand the prepuce is slipped or drawn forward.

By thus putting the vessels on the stretch they are relieved from the pressure of the stricture, and the blood and infiltrated fluids are readily allowed to pass back beyond it, when the glans is easily pushed through the constricted prepuce into its proper position.

After traction and compression of the glans has been continued one minute, the attempt may be made to reduce it, and the first effort will generally succeed, though the grasp is not to be loosened but the pressure is to be continued and the glans forced backward, the right thumb and finger drawing the prepuce forward at the same time.

If the first attempt should fail, repeat the effort according to directions, and success is certain.

If there is a tendency to return of the stricture, instruct the patient to pour a stream of cold water on the glans every fifteen minutes, and to hold it in the normal position until the tendency is overcome.

If the organ is much inflamed and painful to the touch, it would be well enough for the patient to take a good "slug" of whisky before commencing the operation.

After treatment, the cold effusion and arnica dressing may be used for a few days, or until the inflammation subsides.

I have succeeded in reducing several very obstinate cases in less than five minutes by this method.

TREATMENT OF ORCHITIS.

I have tried blistering over the saphenous opening with nitrate of silver, in both acute and chronic orchitis, with the most satisfactory results.

I use in connection with this, active catharsis or emeto catharsis in the beginning of the treatment, and repeat both the blistering and cathartics in three or four days or a week if the case requires it.

As a dressing to the diseased testicle, equal parts vinegar and a strong solution of chloride of sodium, ice cold, acts well.

The patient to be kept in a recumbent position for two or three days, and to wear a suspensory bandage for two or three weeks.

I have never tried the fly-blister, but presume it would act equally as well as the nitrate of silver.

DR. TOM B. DORA.

---

***Petroleum in Dry Rot.***

According to Herbst, petroleum may be applied with excellent advantage in the extirpation of the dry rot, it being only necessary to paint the surface of wood thus affected with the petroleum. A solution of carbolic acid, however, answers the same purpose and involves much less danger from fire.—*Agr. Report.*

